# Timing of Cholecystectomy After Acute Biliary Severe Pancreatitis: A Systematic Review—Time for A Clinical Trial? –

**DOI:** 10.1002/jgh3.70368

**Published:** 2026-04-17

**Authors:** Carlos Eduardo Rey Chaves, Juan Pablo Diaz Amarís, Maria Alejandra Diaz Tarazona, Laura Sarmiento, Danny Conde, Isabella Alarcón, David Gómez Garnica

**Affiliations:** ^1^ Estudiante de Posgrado Cirugía General, Pontificia Universidad Javeriana Facultad de Medicina Bogotá Colombia; ^2^ Médica General, Pontificia Universidad Javeriana Facultad de Medicina Bogotá Colombia; ^3^ Cirujano General – Univerisdad el Rosario Bogotá Colombia; ^4^ Estudiante de Pregrado, Medicina. Pontificia Universidad Javeriana Facultad de Medicina Bogotá Colombia; ^5^ Cirujano General, Pontificia Universidad Javeriana Facultad de Medicina Bogotá Colombia

**Keywords:** cholecystectomy, outcomes, severe acute pancreatitis, timing

## Abstract

**Background:**

There is a lack of evidence about the optimal timing for cholecystectomy in patients with severe acute pancreatitis. The discussion centers on evaluating the benefits of performing early versus delayed cholecystectomy, looking for better outcomes. This systematic review aimed to assess the postoperative outcomes after cholecystectomy in patients with severe pancreatitis regarding the timing of the surgery.

**Methods:**

A systematic review was conducted, and the inclusion criteria included original, peer‐reviewed retrospective or prospective studies involving humans > 18 years old with acute severe pancreatitis due to gallstone disease who underwent laparoscopic cholecystectomy. Newcastle Ottawa score was used to perform quality and bias assessment. The search was conducted from January 2004 to December 2023 within Pubmed, EMBASE, and Scopus.

**Results:**

A total of 1198 studies were found; after screening for duplicates, eligibility criteria and conducting quality assessment, three articles met the inclusion criteria and were included in the systematic review, involving a total of 598 patients. Early cholecystectomy (Defined < 15 days or 6 weeks) was performed in 30.10% (*n* = 179) and delayed cholecystectomy in 70.06% (*n* = 419), with a morbidity rate of 33% (*n* = 60) and 20% (*n* = 86), respectively. Mortality was most frequent when cholecystectomy was performed earlier (2.39% *n* = 10 vs. 0.23% *n* = 1).

**Conclusion:**

Despite limitations regarding the small sample size and the small amount of research published to date, this systematic review suggests that there is a higher risk of morbidity and mortality in patients with severe pancreatitis undergoing early cholecystectomy compared to those undergoing delayed cholecystectomy.

## 1 Introduction

Acute pancreatitis (AP) is an acute inflammatory process of the pancreatic tissue, and it is well known that gallstones and bile sludge are the most common entities related to AP, accounting for at least 50%–60% of the cases; nevertheless, there are other possible causes, such as alcohol abuse, hypertriglyceridemia reaching 25% and 5%, respectively [1, 2].

AP evolution is self‐limited and with a mild course in approximately 85% of the cases with a mortality range between 5% and 15%; however, in some cases, evolution can progress with local or systemic complications, increasing mortality up to 40% in some case series [1, 2, 3, 4]. According to the evidence, the recurrence rate of biliary events after the first episode of AP is up 33%; consequently, cholecystectomy is a cornerstone in treating acute biliary pancreatitis, and it is required to reduce the biliary episodes after AP [5, 6]—the appropriate timing of cholecystectomy after AP is debated and is related to the severity of the disease.

Early cholecystectomy benefits are described in the available literature, and it reduces the recurrence of biliary events (choledocholithiasis, pancreatitis, cholangitis, and cholecystitis); for example, the PONCHO trial reveals that same‐admission cholecystectomy minimizes the recurrence of biliary events and acute pancreatitis without increasing postoperative morbidity or mortality compared with delayed cholecystectomy [6]. Thus, the timing of cholecystectomy is well described in patients with a mild course of disease [6].

Nevertheless, there is a lack of literature on moderately severe and severe cases of AP. The current guidelines do not have enough evidence to support strong recommendations due to the controversial findings, the heterogeneity of the population, and the broad spectrum of clinical presentation of severe pancreatitis, including organ failure with or without local complications such as peripancreatic sterile collections or necrotizing infected pancreatitis [7, 8, 9, 10, 11].

There is no homogeneous definition regarding the timing of cholecystectomy, with reported timeframes ranging from during the same admission to 15 days, 30 days, or even more than 6 weeks post‐admission [6, 7, 8, 9, 10, 11]. Additionally, endoscopic procedures such as papillotomy or biliary stenting before cholecystectomy could impact the course of acute pancreatitis and influence the postoperative outcomes in terms of morbidity, mortality, and recurrence of biliary events [6, 7, 8, 9, 10, 11].

There is a significant gap in the literature regarding this topic. Establishing the appropriate timing for cholecystectomy in patients with severe pancreatitis is crucial to improving postoperative outcomes, including morbidity, mortality, and a major issue: The recurrence of biliary events after severe pancreatitis. This systematic review aims to evaluate the evidence regarding the timing of cholecystectomy in patients with AP classified as severe with or without local complications.

To guide this systematic review, the following PICO strategy was employed:

P Patients with acute severe biliary pancreatitis.

I Cholecystectomy.

C Timing of cholecystectomy.

O Postoperative outcomes.

## 2 Materials and Methods

### 2.1 Search Strategy

This systematic review was reported according to the Preferred Reporting Items for Systematic Review and Meta‐Analysis (PRISMA) Statement [12]. This protocol was registered on PROSPERO under registration number CRD420250631698, and the AMSTAR2 (A Measurement tool to assess systematic reviews) guidelines were consulted and followed. We aimed to evaluate the postoperative outcomes after cholecystectomy in patients with severe pancreatitis regarding the timing of the surgery. The research strategy included the following terms: *“Cholecystectomy”* OR “Cholecystectomies” OR “Cholecystectomy, laparoscopic” OR “Cholecystectomy AND laparoscopic” OR “Laparoscopic cholecystectomy” *AND “Severe” AND “pancreatitis”* OR *“Necrotizing pancreatitis”* OR “Acute necrotizing pancreatitis” OR “*Severe pancreatitis*.” The search was conducted across Pubmed, EMBASE, and Scopus databases for studies published from January 2004 to December 2023.

### 2.2 Selection Criteria

The study inclusion criteria included original, peer‐reviewed retrospective or prospective studies published in english involving human participants > 18 years old with acute severe pancreatitis caused by gallstone disease who underwent laparoscopic cholecystectomy.

Exclusion criteria included letters, case reports, commentaries, conference abstracts, reviews, meta‐analyses, and studies of poor methodological quality.

### 2.3 Data Extraction

Two independent reviewers performed a systematic literature search for studies published between January 2004 and December 2023 in the PubMed, EMBASE, and Scopus databases. After removing duplicates, the reviewers screened the titles and abstracts of the remaining studies to identify those potentially eligible. Full texts of these studies were then retrieved and assessed for inclusion based on the predefined criteria. A third reviewer was included to resolve any discrepancies. Data extraction was performed using a predefined Excel spreadsheet where key information was tabulated and summarized.

Data extracted includes demographic and clinical characteristics, the timing of cholecystectomy in each study, endoscopic retrograde cholangiopancreatography use, and local complications such as pancreatic pseudocyst and infected necrosis of peripancreatic collections. Additionally, the treatment required for local complications (endoscopic/percutaneous or surgically treated) and the presence of severe complications such as intestinal ischemia or abdominal compartmental syndrome were recorded. Other extracted variables included overall postoperative morbidity, bile duct injury, and complications classified as Clavien Dindo grade > III, as well as postoperative mortality, overall length of stay, and readmission rate. Relevant data from all the studies were extracted and reported.

### 2.4 Assessment of Risk of Bias

Two evaluators used the Newcastle Ottawa score for systematic reviews to evaluate the risk of bias and applicability of the included studies; in case of any discrepancy, a third reviewer was included. (Table 1).

**TABLE 1:** Risk of bias—The Newcastle Ottawa Scale of assessing the quality of non‐randomized studies.

Study	Selection (0–3)	Comparability (0–2)	Outcome (0–3)	Total
Di Martino et al. 2023 [13]	3	2	3	8—Included
Nealon et al. 2004 [14]	2	1	3	6—Included
Hallensleben et al. 2021 [15]	3	2	3	8—Included
Fong et al. 2015 [16]	1	1	2	4—Excluded

### 2.5 Assessment of Heterogeneity

The heterogeneity of the included studies was assessed, identifying differences in both the interventions and the reported outcomes. As a result of these discrepancies, a meta‐analysis was not considered appropriate, and the evidence was synthesized narratively.

## 3 Results

### 3.1 Study Selection

**FIGURE 1:**
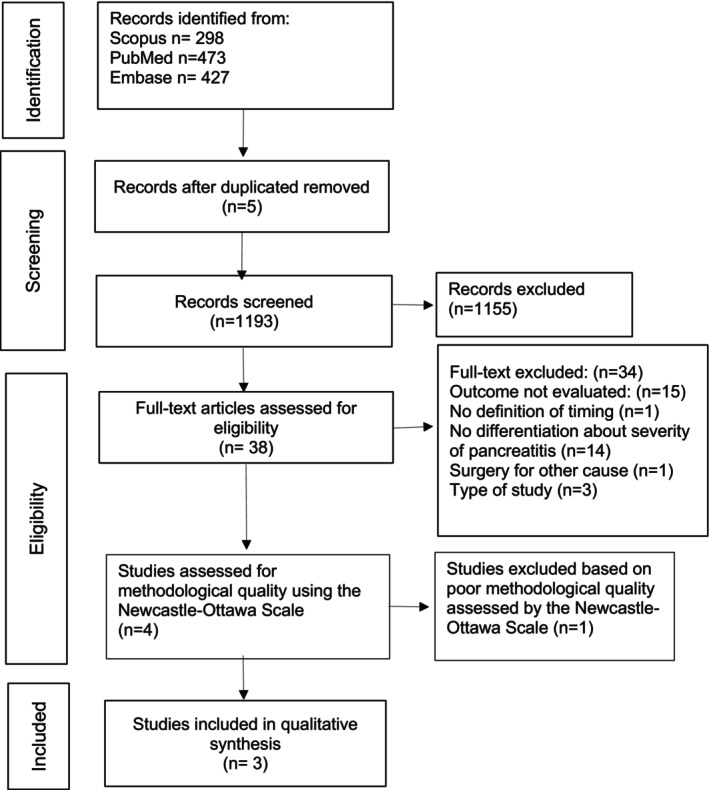
Study flow diagram.

A total of 1198 references were obtained from PUBMED, EMBASE, and SCOPUS, excluding duplicates. After abstract and title revision, 38 articles were selected for a full‐text analysis. Then, 34 articles were excluded (See Figure 1), and finally, four studies were evaluated; nevertheless, one study was excluded due to poor quality assessment. Figure 1 displays a flow chart summarizing the research process.

Finally, three studies were included in the systematic review [13, 14, 15]; one was a multicenter retrospective study, including 42 countries; the other evaluated the North American population (USA), and the last was performed in the Netherlands.

### 3.2 Demographic and Clinical Characteristics

Our data synthesis included 598 patients with severe acute biliary pancreatitis. The Demographic and clinical characteristics evidenced a mean age of 51.13 years; most of the population was male in 53.84% (*n* = 322) of the cases. An ASA score>III was reported in 6.68% (*n* = 40); however, one study did not report this variable. Comorbidities were documented in only one study, with type 2 diabetes mellitus being the most common, affecting 4.34% (*n* = 26) of the patients. In 53.34% (*n* = 319) of cases, local complications were observed, with infected necrosis in 42.31% (*n* = 135). In 37.12% (*n* = 222) of the cases, an endoscopic or percutaneous approach was preferred for local complications, and in 4.34% (*n* = 26) of the cases, surgical necrosectomy was performed. One study reported severe complications related to pancreatitis that required surgical intervention, such as intestinal ischemia or abdominal hypertension leading to compartment syndrome and was evidenced in 3.51% (*n* = 21) of the entire population.

### 3.3 Timing of Cholecystectomy

Another critical topic is the lack of a consistent definition for early versus late cholecystectomy. Our analysis revealed that the timing of cholecystectomy is not standardized across the available evidence, and all studies differ. One study defined early cholecystectomy as < 15 days of admission and delayed cholecystectomy if performed > 15 days of admission [13]. Another study categorized early cholecystectomy as occurring < 6 weeks from admission, even in the presence of local complications, and delayed cholecystectomy if performed > 6 weeks from admission [14]. The third study evaluated different time frames (4, 6, 8, 10, and 12 weeks) and analyzed the impact of ERCP combined with sphincterotomy regarding the recurrence of biliary events.

### 3.4 Postoperative Outcomes and Related Factors for Morbidity and Mortality

In terms of morbidity and mortality according to the timing of the intervention, early cholecystectomy was performed in 30.10% (*n* = 179) and delayed cholecystectomy in 70.06% (*n* = 419), with a morbidity rate of 33% (*n* = 60) and 20% (*n* = 86), respectively. Mortality was most frequent when cholecystectomy was performed earlier (2.39% *n* = 10 vs. 0.23% *n* = 1). Additionally, the postoperative length of stay was longer in the early cholecystectomy group, with a mean of 15.5 days compared to 7,75 days in the delayed group. Readmission rates due to biliary events were assessed in only one study, which found that readmissions were most frequent in the delayed cholecystectomy group when there was a delay of more than 8 weeks. (See Table 2).

**TABLE 2:** Summary of the included studies.

	Study (ref.) year	Number of patients	Study period	Timing of cholecistectomy	Severe complications requiring surgery	Post cholecystectomy mortality	Post cholecystectomy morbility	Bile duct injury	Readmission rate	Recurrence of biliary events	Relevant Data
1	Di Martino et al. 2023 [13]	105	January 1, 2019 and December 31, 2020	Delayed Cholecystectomy > 14 days/Early cholecystectomy < 14 days	*n* (%)	*n* (%)	*n* (%)	*n* (%)	*n* (%)	Does not evaluate	Severe complications requiring surgery (AOR,33.64;95% CI, 3.19–354.73;*p* = 0.003) and Not having and ERCP (AOR, 0.06; 95% CI, 0.001–61.69; *P* = 0.01) are associated with morbidity
					7 (10)/14 (44)	0 (0)/10 (32)	9 (17)/13 (48)	1 (2)/0 (0)	15 (26)/5 (19)		
				73/32							Age (AOR, 1.12; 95% CI, 1.02–1.36; *P* = 0.03), ASA Score (AOR, 5.91; 95% CI, 1.06–32.78; *P* = 0.04), and severe complications requiring surgery (AOR, 50.04; 95% CI, 2.37–1058.01; *P* = 0.01) are associated with mortality
2	Nealon et al. 2004 [14]	187	1987–2004	Early Cholecystectomy < 6 weeks/Delayed Cholecystectomy > 6 weeks	NA	*n* (%)	*n* (%)	NA	N	Does not evaluate.	Cholecystectomy should be delayed after an episode of severe pancreatitis with local complications at least 6 weeks
				78/109		1 (1.3)/0 (0)	34 (44)/6 (5.5)		49/NA		
3	Hallensleben et al. 2021 [15]	248	Between 2008 and 2015	Overall time analysis: median 103 days (46–222)	NA	NA	*n* (%)	*n* (%)	*n* (%)		Delayed cholecystectomy > 8 weeks is related to an increased risk of biliary events
				< 4 weeks: 25			< 4 Weeks: 3 (12) > Weeks 19 (11)	< 4 Weeks: 1 (4) > 4 Weeks 6 (4)	< 4 Weeks: 2 (8) > 4 Weeks 54 (24)	Risk of biliary events was lower if cholecystectomy was performed < 8 weeks (RR 0.14 P 0.02 95% CI 0.02–0.99)	There is no increased risk of morbidity or mortality over time
				< 6 weeks: 45			< 6 weeks 3 (7) > 6 Weeks 19 (13)	< 6 weeks 1 (2) > 6 Weeks 6 (4)	< 6 weeks 9 (20) > 6 Weeks 47 (23)		There is no evidence that ERCP plus spinchterotomy influence the recurrence of biliary events over the time
				< 8 weeks: 67			< 8 Weeks 7 (10) > 8 Weeks 15 (12)	< 8 Weeks 1 (2) > 8 Weeks 6 (5)	< 8 Weeks 12 (18) > 8 Weeks 44 (24)		
				< 10 weeks: 81			< 10 Weeks 8 (10) > 10 Weeks 14 (13)	< 10 Weeks 1 (1) > 10 Weeks 6 (6)	< 10 Weeks 13 (16) > 10 Weeks 43 (26)		
				< 12 weeks: 88			< 12 Weeks 9 (10) > 10 Weeks 10 (10)	< 12 Weeks 1 (1) > 10 Weeks 6 (6)	< 12 Weeks 13 (15) > 10 Weeks 43 (27)		

Some additional topics were discussed in the evaluated studies. Clinical characteristics like the presence of severe complications requiring surgery (AOR, 33.64; 95% CI, 3.19–354.73; *p* = 0.003) and not having an endoscopic retrograde cholangiopancreatography (ERCP) (AOR, 0.06; 95% CI, 0.001–61.69; *p* = 0.01) were associated with the presence of any complication; for mortality, characteristics like age (AOR, 1.12; 95% CI, 1.02–1.36; *p* = 0.03), ASA Score (AOR, 5.91; 95% CI, 1.06–32.78; *p* = 0.04), and the presence of severe complications requiring surgery (AOR, 50.04; 95% CI, 2.37–1058.01; *p* = 0.01) were found to be significant predictors [12, 14, 17].

### 3.5 Recurrence of Bile Events

The recurrence of biliary events after acute severe pancreatitis was also an important topic. According to the evidence, delayed cholecystectomy, when performed after 8 weeks, is associated with an increased risk of biliary events in one study. Additionally, there is no evidence that ERCP plus sphincterotomy influences the recurrence of biliary events over time.

## 4 Discussion

This systematic review suggests that there is a higher risk of morbidity and mortality in patients with severe pancreatitis undergoing early cholecystectomy (EC) compared to those undergoing delayed cholecystectomy (DC). Therefore, treatment should be postponed until the inflammatory process is controlled. However, the lack of high‐quality data makes it challenging to provide a conclusive recommendation.

The timing of cholecystectomy remains a controversial topic with ongoing debate in the literature. According to the Oxford Center of Evidence‐based Medicine, the level of this evidence does not exceed grade 2c [13].

Further prospective studies are needed to clarify this issue. Advocates of EC argue that it leads to a shorter length of hospital stay (LOS), avoids interim biliary events, and prevents recurrent pancreatitis. However, concerns regarding EC are the technical difficulty due to inflammation and some evidence suggesting increased morbidity and mortality [9, 10, 11, 13].

On the other hand, delayed cholecystectomy is related to an increased risk of biliary events, which could reach 33%, and recurrent pancreatitis [18]. In their study, Prashant et al. described 13.3% of biliary events compared to 2% in EC patients [18]. Moreover, Ito and colleagues reported biliary events in 50% of cases within the following four weeks after discharge [19].

Current Japanese and British guidelines recommend early cholecystectomy in mild pancreatitis and delayed cholecystectomy in moderate and severe cases of gallstone pancreatitis until resolution of pulmonary and organic failure [17, 20]. However, there are some issues with these recommendations. First, the waiting time between all the guidelines is unclear, so the “delayed cholecystectomy” time is heterogeneous. In one of the most recent systematic reviews, DC was defined as cholecystectomy done after the resolution of symptoms of pancreatitis or after a specific time interval (after 48 h or 6 weeks). Other studies defined it as more than 4 weeks. We identified in this systematic review a heterogeneity in the definition of early cholecystectomy, with a range variance between less than 15 days and 6 weeks, which is a vast range and makes a homogeneous evaluation difficult [18].

It is also relevant to notice in this systematic review the finding that more than half of the patients presented with local complications (53.34%), and infected necrosis was the most frequent (42.31%). So, the presence of local complications is relevant to evaluate this condition's morbidity and should be evaluated in a subgroup category from the severe cases due to organ failure. IAP/APA guidelines recommend a delayed cholecystectomy after 6 weeks, specifically in cases where a peripancreatic collection exists or when the collection resolves [9, 18].

The recurrence of biliary events needs to be assessed, and it is an outcome evaluated in the timing of cholecystectomy in cases of severe acute pancreatitis. Regarding this topic, Hallensleben et al. [15] assessed the clinical impact of performing ERCP and endoscopic sphincterotomy in the recurrence of biliary events. However, according to their findings, ERCP with endoscopic sphincterotomy does not reduce the risk of biliary events until cholecystectomy (OR 1.40 (95% CI 0.74 to 2.83)). On the other hand, Di Martino et al. [13] findings revealed that in cases of early cholecystectomy, not performing ERCP before surgery should be considered as an independent factor for morbidity after cholecystectomy (AOR 0.06; 95% CI 0.001–61.69; *p* = 0.01) [13].

Based on these findings, the clinical impact of performing ERCP and sphincterotomy in patients with acute severe pancreatitis remains unclear both in terms of the recurrence of biliary events and morbidity related to early or delayed cholecystectomy. The current evidence is insufficient to establish the procedure's benefit in this context. Additionally, there is no homogeneous definition of early or delayed cholecystectomy; this topic requires further study to clarify it and provide appropriate recommendations, based on morbidity, mortality, and recurrence of biliary events.

To the best of our knowledge, this is the first systematic review focused on severe pancreatitis cases undergoing EC and DC. The limitations of our review and possible biases include the small number of studies that met the inclusion criteria, the retrospective nature of the selected studies, and the heterogeneity in some definitions described previously.

Higher‐quality evidence in patients with gallstones and severe pancreatitis needs to be provided to provide robust recommendations. Additionally, the relatively small incidence of this specific condition compared to mild presentations presents challenges for data extraction and increases the risk of bias. A novel randomized clinical trial protocol has been published and is set to begin, aiming to address these uncertainties [16, 21]. Nevertheless, our findings underscore the need for future prospective studies and randomized clinical trials to determine the optimal timing of cholecystectomy in patients with severe acute pancreatitis, regardless of whether they have local acute or chronic complications.

## 5 Conclusion

Based on the current evidence, early cholecystectomy in cases of severe pancreatitis cannot be recommended due to the associated increased risks of morbidity and mortality. Furthermore, cases of severe pancreatitis should be stratified according to the presence of local complications, as these significantly influence the morbidity and mortality associated with ongoing surgical procedures. Future clinical trials are essential to clarify or refute these findings and provide clarity on the optimal timing of cholecystectomy in these patients.

## Funding

The authors have nothing to report.

## Ethics Statement

All procedures performed in studies involving human participants were in accordance with the ethical standards of the institutional and/or national research committee, and with the 1964 Helsinki Declaration and its later amendments or comparable ethical standards.

## Consent

The authors have nothing to report.

## Conflicts of Interest

The authors declare no conflicts of interest.

## Data Availability

The data that support the findings of this study are available on request from the corresponding author. The data are not publicly available due to privacy or ethical restrictions.

## References

[1] M. A. Mederos , H. A. Reber , and M. D. Girgis , “Acute Pancreatitis: A Review,” JAMA 325, no. 4 (2021): 382–390, 10.1001/jama.2020.20317. 33496779

[2] P. A. Banks , T. L. Bollen , C. Dervenis , et al., “Classification of Acute Pancreatitis‐2012: Revision of the Atlanta Classification and Definitions by International Consensus,” Gut 62, no. 1 (2013): 102–111, 10.1136/gutjnl-2012-302779. 23100216

[3] C. E. Forsmark , S. S. Vege , and C. M. Wilcox , “Acute Pancreatitis,” New England Journal of Medicine 375, no. 20 (2016): 17–1981, 10.1056/NEJMra1505202. PMC13220086 27959604

[4] P. G. Lankisch , M. Apte , and P. A. Banks , “Acute Pancreatitis,” Lancet 386, no. 9988 (2015): 4–96, 10.1016/S0140-6736(14)60649-8. 25616312

[5] W. Dai , Y. Zhao , G. L. Du , and R. P. Zhang , “Comparison of Early and Delayed Cholecystectomy for Biliary Pancreatitis: A Meta‐Analysis,” Surgeon 19, no. 5 (2021): 257–262, 10.1016/j.surge.2020.06.012. 32768360

[6] D. W. da Costa , S. A. Bouwense , N. J. Schepers , et al., “Same‐Admission Versus Interval Cholecystectomy for Mild Gallstone Pancreatitis (PONCHO): A Multicentre Randomised Controlled Trial,” Lancet 386, no. 10000 (2015): 26–1268, 10.1016/S0140-6736(15)00274-3. 26460661

[7] A. Leppäniemi , M. Tolonen , A. Tarasconi , et al., “2019 WSES Guidelines for the Management of Severe Acute Pancreatitis,” World Journal of Emergency Surgery 14 (2019): 27, 10.1186/s13017-019-0247-0. 31210778 PMC6567462

[8] M. Yokoe , T. Takada , T. Mayumi , et al., “Japanese Guidelines for the Management of Acute Pancreatitis: Japanese Guidelines 2015,” Journal of Hepato‐Biliary‐Pancreatic Sciences 22, no. 6 (2015): 405–432, 10.1002/jhbp.259. 25973947

[9] Working Group IAP/APA Acute Pancreatitis Guidelines , “IAP/APA Evidence‐Based Guidelines for the Management of Acute Pancreatitis,” Pancreatology 13, no. 4 Suppl 2 (2013): 1–15, 10.1016/j.pan.2013.07.063. 24054878

[10] S. Tenner , J. Baillie , J. DeWitt , S. S. Vege , and American College of Gastroenterology , “American College of Gastroenterology Guideline: Management of Acute Pancreatitis,” American Journal of Gastroenterology 108, no. 9 (2013): 1400–1415, 10.1038/ajg.2013.218. 23896955

[11] W. Sun , L. Y. An , X. D. Bao , et al., “Consensus and Controversy Among Severe Pancreatitis Surgery Guidelines: A Guideline Evaluation Based on the Appraisal of Guidelines for Research and Evaluation II (AGREE II) Tool,” Gland Surgery 9, no. 5 (2020): 1551–1563, 10.21037/gs-20-444. 33224831 PMC7667075

[12] M. J. Page , J. E. McKenzie , P. M. Bossuyt , et al., “The PRISMA 2020 Statement: An Updated Guideline for Reporting Systematic Reviews,” BMJ 372 (2021): 29, n71, 10.1136/bmj.n71. PMC8005924 33782057

[13] M. Di Martino , B. Ielpo , F. Pata , et al., “Timing of Cholecystectomy After Moderate and Severe Acute Biliary Pancreatitis,” JAMA Surgery 158, no. 10 (2023): e233660, 10.1001/jamasurg.2023.3660. 37610760 PMC10448376

[14] W. H. Nealon , J. Bawduniak , and E. M. Walser , “Appropriate Timing of Cholecystectomy in Patients Who Present With Moderate to Severe Gallstone‐Associated Acute Pancreatitis With Peripancreatic Fluid Collections,” Annals of Surgery 239, no. 6 (2004): 741–749, 10.1097/01.sla.0000128688.97556.94. 15166953 PMC1356283

[15] N. D. Hallensleben , H. C. Timmerhuis , R. A. Hollemans , et al., “Optimal Timing of Cholecystectomy After Necrotising Biliary Pancreatitis,” Gut 71, no. 5 (2022): 974–982, 10.1136/gutjnl-2021-324239. 34272261

[16] Z. V. Fong , M. Peev , A. L. Warshaw , et al., “Single‐Stage Cholecystectomy at the Time of Pancreatic Necrosectomy Is Safe and Prevents Future Biliary Complications: A 20‐Year Single Institutional Experience With 217 Consecutive Patients,” Journal of Gastrointestinal Surgery 19, no. 1 (2015): 32–37, 10.1007/s11605-014-2650-x. 25270594

[17] Y. Kimura , T. Takada , Y. Kawarada , et al., “JPN Guidelines for the Management of Acute Pancreatitis: Treatment of Gallstone‐ Induced Acute Pancreatitis,” Journal of Hepato‐Biliary‐Pancreatic Surgery 13 (2006): 56–60. 16463212 10.1007/s00534-005-1052-6 PMC2779396

[18] J. Prasanth , M. Prasad , S. J. Mahapatra , et al., “Early Versus Delayed Cholecystectomy for Acute Biliary Pancreatitis: A Systematic Review and Meta‐Analysis,” World Journal of Surgery 46, no. 6 (2022): 1359–1375, 10.1007/s00268-022-06501-4. 35306590

[19] K. Ito , H. Ito , and E. E. Whang , “Timing of Cholecystectomy for Biliary Pancreatitis: Do the Data Support Current Guidelines?,” Journal of Gastrointestinal Surgery 12 (2008): 2164–2170. 18636298 10.1007/s11605-008-0603-y

[20] Working Party of the British Society of Gastroenterology , “Association of Surgeons of Great Britain and Ireland, Pancreatic Society of Great Britain and Ireland. UK Guidelines for the Management of Acute Pancreatitis,” Gut 54v1 (2005): 16, 10.1136/gut.2004.057059.

[21] C. Ramírez‐Giraldo , D. Conde Monroy , J. A. Daza Vergara , A. Isaza‐Restrepo , I. Van‐Londoño , and L. Trujillo‐Guerrero , “Timing of CHolecystectomy in Severe PAncreatitis (CHISPA): Study Protocol for a Randomized Controlled Trial,” BMJ Surgery, Interventions & Health Technologies 6, no. 1 (2024): e000246, 10.1136/bmjsit-2023-000246. PMC10921534 38463464

